# Promoter-Associated RNAs Regulate HSPC152 Gene Expression in Malignant Melanoma

**DOI:** 10.3390/ncrna2030007

**Published:** 2016-06-30

**Authors:** Hamutal Bonen, Nitzan Kol, Noam Shomron, Raya Leibowitz-Amit, Luca Quagliata, Thomas Lorber, Yechezkel Sidi, Dror Avni

**Affiliations:** 1 Laboratory of Molecular Cell Biology, Centre for Cancer Research and Department of Medicine C, Chaim Sheba Medical Centre, Tel-Hashomer 52621, Israel; hamu_b@yahoo.com; 2 Functional Genomics Laboratory, Sackler Faculty of Medicine, Tel Aviv University, Tel Aviv, Israel; nitzankol@gmail.com (N.K.); nshomron@post.tau.ac.il (N.S.); 3 Institute of Oncology, Sheba Medical Center, Tel Hashomer 52621, Israel; Raya.Leibowitz-Amit@sheba.health.gov.il; 4 Molecular Pathology Unit, Institute of Pathology University Hospital Basel, CH-4031 Basel, Switzerland; Luca.Quagliata@usb.ch (L.Q.); Thomas.Lorber@usb.ch (T.L.)

**Keywords:** Promoter-Associated RNA, HSPC152, TYR, Melanoma

## Abstract

The threshold of 200 nucleotides (nt) conventionally divides non-coding RNAs (ncRNA) into long ncRNA (lincRNA, that have more than 200 nt in length) and the remaining ones which are grouped as “small” RNAs (microRNAs, small nucleolar RNAs and piwiRNAs). Promoter-associated RNAs (paRNAs) are generally 200–500 nt long and are transcribed from sequences positioned in the promoter regions of genes. Growing evidence suggests that paRNAs play a crucial role in controlling gene transcription. Here, we used deep sequencing to identify paRNA sequences that show altered expression in a melanoma cell line compared to normal melanocytes. Thousands of reads were mapped to transcription start site (TSS) regions. We limited our search to paRNAs adjacent to genes with an expression that differed between melanoma and normal melanocytes and a length of 200–500 nt that did not overlap the gene mRNA by more than 300 nt, ultimately leaving us with 11 such transcripts. Using quantitative real-time PCR (qRT-PCR), we found a significant correlation between the expression of the mRNA and its corresponding paRNA for two studied genes: *TYR* and *HSPC152*. Ectopic overexpression of the paRNA of *HSPC152* (designated paHSPC) enhanced the expression of the *HSPC152* mRNA, and an siRNA targeting the paHSPC152 decreased the expression of the *HSPC152* mRNA. Overexpression of paHSPC also affected the epigenetic structure of its putative promoter region along with effects on several biologic features of melanoma cells. The ectopic expression of the paRNA to *TYR* did not have any effect. Overall, our work indicates that paRNAs may serve as an additional layer in the regulation of gene expression in melanoma, thus meriting further investigation.

## 1. Introduction

The human genome project revealed that approximately 90% of the sequence is actively transcribed, but only 1%–1.5% is translated to protein products [1]. The transcriptional products which are not transcribed are referred to as non-coding RNAs (ncRNA). The most commonly used classification is based on RNA length: the dividing line is set at 200 nucleotides (nt). Longer RNAs are considered to be long ncRNAs (lincRNA), and shorter RNAs are considered short ncRNA (microRNAs, small nucleolar RNAs and piwi-interacting RNA) [2,3]. Promoter-associated RNAs (paRNAs) are generally 200–500 nt long. paRNAs were found in yeast and *Arabidopsis* and span the size of ≈250–500 nucleotides (nt) [3,4]. Napoli et al. showed the presence of low copy RNA transcripts in the region from −400 to +120 (520 nt) relative to the transcription start site (TSS) of the c-myc gene [5]. Seila et al. showed divergent transcription around active promoters and active TSS, both in abundance and size. The low abundance RNA are around 500 nt [6]. They are transcribed from sequences positioned in the promoter regions of genes. Those lncRNAs were first identified by Han et al. [7] and described as “sense-stranded RNA transcripts corresponding to the known promoter region” that may serve as a target for siRNAs targeting promoter regions and inducing transcriptional gene silencing.

A second and potentially overlapping class of paRNAs are transcription start site-associated RNAs, that are 20–90 nt long and localized within −250 to +50 of TSSs. A third class of paRNA are transcription initiation RNAs which are 18 nt in length and have their highest density just downstream of TSSs [3,4,8]. LincRNAs are known to have dynamic expression patterns in different cell types, tissues and differentiation stages [9]. These transcripts appear in low copy number per cell, are often poorly conserved throughout evolution and are very unstable [1]. Their functional importance is far from being understood, but an increasing number of studies have shown their ability to regulate diverse functions such as X chromosome silencing [10], pluripotency [11] and epigenetic gene regulation [12]. LincRNAs can be classified, based on their genomic position in relation to protein-coding genes, as intronic or intergenic and also in accordance with their orientation (in respect to protein-coding transcripts) as sense or antisense [13].

PaRNAs are lincRNAs with sequence complementarity to parts of gene promoters. A few studies suggested that paRNAs promote silencing of gene transcription from their cognate promoter [7,14,15], whereas only one work suggested that paRNA promotes transcription of the c-myc gene [5]. Recent studies have found that paRNAs serve as scaffolds for antisense transcripts that regulate gene transcription as reviewed in [8,16]. In this current research, we set out to identify and isolate new paRNAs from an in vitro model of melanocyte melanoma and assess the association between paRNA expression and transcription of the cognate gene.

## 2. Materials and Methods

### 2.1. Cell Lines and Melanoma Biopsies 

Melanoma cell lines were generated directly from metastatic melanoma lesions of patients at the surgical branch of the National Institute of Health (NIH, Bethesda, MD, USA) (mel526, mel624, mel33B1 [14,15]) or at the Ella Institute for Melanoma Research at the Sheba Medical Center (Tel-Hashomer, Israel) (014mel, 15AY) [15]. MNT-1 cell lines were generously given by Dr. Patrizio Giacomini of the immunology lab at the “Regina Elena” National Cancer institute Rome, Italy [16].

Melanoma lines were grown in Dulbecco’s Modified Eagle Medium containing 10% fetal bovine serum (FBS), 1% l-glutamate, 1% penicillin-streptomycin mixture (P/S mix, full medium) and 2.5% HEPES solution. All materials were purchased from Biological Industries Ltd. (Beit Haemek, Israel).

Three different batches of normal human epidermal melanocytes (NHEM) cell line were purchased from PromoCell (c-12400; PromoCell, Heidelberg, Germany) and grown in Melanocyte Growth Medium containing 0.6% supplement mix (PromoCell, cat No. c-24010 and c-39415, respectively), and 1% P/S mix.

MelST, melST-R and melST-M-transformed melanocytes [17] were generously donated by Dr. Robert Weinberg’s lab (Whitehead Institute for Biomedical research Cambridge, MA, USA) and were grown in full medium. In brief, the melST line, immortalized but non-malignant melanocytes, was developed through the transfection of NHEM with the SV40ER and hTERT cDNA. The two additional melST sub-lines were created using the RAS protein (melST-R) or the active form of the c-Met receptor (melST-M) and both demonstrate a fully transformed malignant phenotype [17].

All cell lines were grown at 37 °C, 8% CO_2_ and 99% humidity.

All of the biopsy samples were a gift from Dr. Luca Quagliata, at the Molecular Pathology Unit, Institute of Pathology, University Hospital Basel, Switzerland.

The research use of the clinical specimens was approved (N.310/10) by the ethical committee of the University Hospital of Basel, Switzerland. RNA samples were sent to us on dried ice and incubated at −80 °C until used.

### 2.2. RNA Purification and Enhancement 

Total RNA was extracted from cells using the Total RNA Purification Kit (cat# 17200, Norgen Biotek Corp, Thorold, ON, Canada). For the deep sequencing project and for paRNA detection, cells were harvested using Trizol^®^ reagent (Invitrogen^TM^, Thermo-Fisher Scientific Inc., Waltham, MA, USA). All purifications were performed according to the manufacturer’s protocols. Reverse transcription-polymerase chain reaction (RT-PCR) was performed using the PrimeScript^TM^ RT reagent Kit (cat. No. RR037, TaKaRa Bio Inc., Otsu, Japan), using both random hexamer and OligodT. The SYBR Green method was used to detect genes’ mRNA and paRNAs. An amount of 1 µL or 2 µL (gene expression or paRNA expression, respectively) of RT product was amplified in a 96-well plate containing 5 µL Power SYBR^®^ Green Master mix (Applied Biosystems Inc., Foster City, CA, USA), and 5 pmol primers. Plates were incubated on the ABI 7900HT thermocycler (Applied Biosystems Inc., Foster City, CA, USA) for 45 cycles. Primers sequences are detailed in Table 1.

For mRNA-paRNA detection and correlation assay, 600 ng of DNA-free RNA (cleaned with the Turbo DNA-free Kit, (Ambion Thermo-Fisher Scientific Inc., Waltham, MA, USA) were used. Using the same threshold for all runs for each sample, the average cycle threshold (CT) was calculated for at least three different runs, and the correlation between the gene’s mRNA and paRNA was measured. On the mRNA assay alone, different samples were normalized using the RPLPO gene.

### 2.3. Deep Sequencing (NGS)

All the procedures for the deep sequencing (deep-seq) (also known as Next Generation Sequencing, or NGS) were performed by the *Functional Genomics Laboratory*, at Tel-Aviv University, Tel-Aviv, Israel.

Total RNA from NHEM cells and 014mel melanoma cell line were used for deep-seq. The first enrichment for potential paRNA was performed by leaving out RNAs corresponding to rRNAs and tRNA. Then, by size separation using gel electrophoresis, only RNAs corresponding to 200–500 nt were extracted for further treatment and analysis. Illumina’s Directional mRNA-Seq Sample Preparation protocol was used with one adjustment: After the RNA gel extraction, neither RNA purification (Poly-A “fishing”) nor RNA fragmentation were used and samples were directly treated with polynucleotide Kinase and Antarctic Phosphatase. Quality control analysis was performed on all libraries using Agilent Technologies 2100 Bioanalyzer (Santa Clara, CA, USA).

### 2.4. Cloning and Plasmids

PTER+ plasmid was used for all cloning [18], except for paTYR sense, where pcDNA3 plasmid was used. Genomic fragments matching the paRNAs were amplified by PCR, using the PhusionTM master mix (cat# F-531S, Finnzymes OY, 02150 Espoo, Finland) with 10 pmol of the following primers:

HSPC152 F-5′-CGACGGTGTTAGGCGC 3′
HSPC152 R-5′-CGGGTACCTGGAGGCG 3′
TYR F-5′-CATTTGCAAGGTCAAATCATC 3′
TYR R-5′-AGTACAAAACAGCCAGGAGC 3′

PCR fragments were cloned using the TOPO TA Cloning Kit into pCRII-TOPO plasmid (cat# 450640, Invitrogen^TM^). For sense-oriented paHSPC, *Eco*RV and *Hin*dIII restriction enzymes were used to clone the segment into pTER plasmid (cat# R01955, R01045, respectively). *Eco*RV and *Bam*HI (cat# R01365) restriction enzymes were used for antisense-oriented paHSPC and paTYR, and sense-oriented paTYR was cut from TOPO paTYR using *Eco*RI restriction enzyme (cat# R0101S) and cloned into pcDNA3 plasmid. TYR sense/antisense orientation was determined using sequencing with pcDNA3.1-F primer (5′-CTCTGGCTAACTAGAGAAC-3′, Hylab Ltd., Rehovot, Israel). All enzymes were purchased from New England Biolabs^®^ Inc. (Ipswich, MA, USA).

### 2.5. siRNA Transfection

The siRNA primer was 5′-ACACAUUUUACUCCUACACAGGCdTdT-3′ and was ordered from Sigma-Aldrich Israel Ltd. (Rehovot, Israel). Transfection of siRNA was performed with X-tremeGENE Transfection Reagent (Roche, CH-4070, Basel, Switzerland).

### 2.6. Plasmid Transfection

All plasmid transfections were performed using Lipofectamin 2000 reagent by Invitrogen^TM^, according to the manufacturer′s protocols. For 24 h prior to transfection, cells were seeded in 6-well plates and grown to 60% confluence. Cell growth medium was replaced shortly before transfection to cell growth medium without P/S mix. Four hours post-transfection, the medium was replaced with full growth medium. Incubation time was set on 0–72 h, depending on the assay performed.

Melanoma 014mel sub-lines containing sense or antisense paRNA were created using 300 µg/mL of the selective antibiotic Zeocin or 2 mg/mL neomycin.

### 2.7. Chromatin-Immunoprecipitation (ChIP) Assay

The 014mel melanoma cells, stably expressing sense or antisense paRNA, were subjected to a ChIP assay using a modified protocol as previously described [18]. In short, cells were seeded in 145 × 20 mm plates and grown to 80%–90% confluence. Chromatin cross-linking was performed using 1% formaldehyde followed by quenching with 0.125 M glycine. Cells were scraped from plates, washed and re-suspended in 1% sodium dodecyl sulfate (SDS)-containing lysis buffer. Lysed cells were sonicated with five sets of 10 s each, to produce DNA segments of ≈1000 bp. Subsequently, 20–25 µg of segmented DNA were taken for overnight immune precipitation at 4 °C using antibodies (Millipore, Billerica, MA, USA) for the three most common histone methylations: H3K4me3, H3K9me3 and H3K27me3, which characterize inactive chromatin. As a control, IgG antibodies were used.

Quantitative analysis was performed by real-time PCR with the same primers for the paRNA detection, using 014mel cells stably expressing an empty plasmid as control.

### 2.8. Cytosine Methylation

The 014mel cells stably expressing either empty plasmid or sense/antisense-oriented paHSPC plasmid were seeded in 6-well plates, in triplicate, and grown to ≈60% confluence. Cells were then harvested from the wells and DNA was purified using the ArchivePure DNA Cell/Tissue Kit (ref #2900267, 5 Prime Inc. Gaithersburg, MD, USA). After DNA purification, the amount of 500 ng was taken from each sample for bisulfite treatment using the EZ-DNA Methylation-Gold Kit (cat. No D5005, the Epigenetics Company, Zymo Research, Irvine, CA, USA) and 4 µL/tube from converted samples were taken for PCR amplification using two sets of primers:

BS F3: 5′-TTGTATATGATTTGTATTTTACGAAGAA
BS R4: 5′-AATAAAAAACCTATCGAATCACG

And,

BS F6: 5′-AGCGGAGGACGATTTTTT
BS R6: 5′-CATGCGAGCTCAGCAGA

For gel detection, 2 µL of PCR products were taken while the rest of the sample was cleaned using the GeneJET Gel Extraction and DNA Cleanup Micro Kit (cat. No K0831, Thermo Scientific Inc., Waltham, MA, USA). Clean PCR products were sent for sequencing analysis and paHSPC expressing a cell sequence was compared to control cells using the BioEdit alignment editor software (North Carolina State University, Ranleigh, NC, USA).

## 3. Results

### 3.1. Screening for paRNAs Differentially Expressed in Normal Melanocytes and Melanoma Cells by Deep-Sequencing Analysis

Our first aim was to search for paRNAs which are differentially expressed in melanoma in comparison to normal melanocytes. To address this question, we purified RNA from normal melanocytes and from the melanoma cell line 014mel [19]. Since the expected amount of paRNA is low compared to total cellular RNA and since we did not know whether these paRNAs contain a poly-A tail, we decided to enrich these RNAs by depletion of the rRNA using the Ribo-Zero (Illumina, San Diego, CA, USA) rRNA Removal Core Kit. We focused on ncRNAs at a length of 200–500 bp, because paRNAs were previously characterized to be of this length [20].

**Figure 1:**
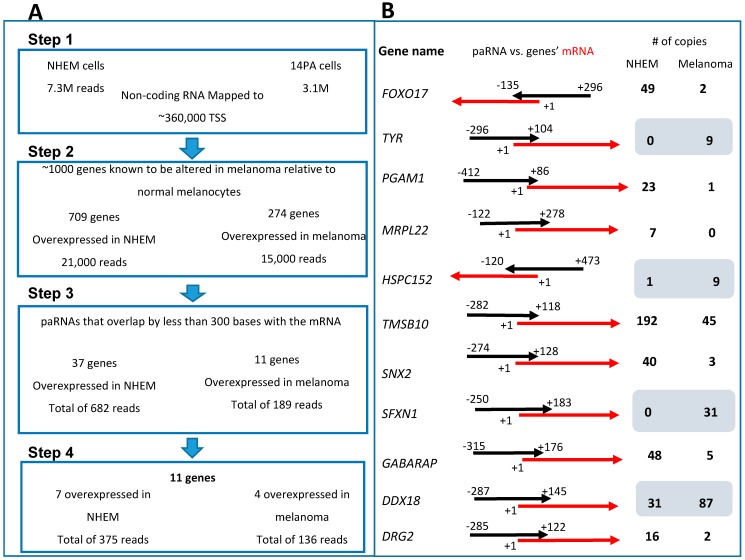
(**A**) Step 1: Several million long non-coding RNAs (lincRNAs) were detected by deep sequencing for each cell line and 10.4 × 10^6^ reads were mapped to 360,000 transcription start sites (TSS). Step 2: A list of melanoma differential-expressing genes was used to detect those lincRNA that mapped to promoters of genes whose expression is altered in melanoma, leaving around 36,000 reads in 983 genes. Step 3: From this list we selected only those promoter-associated RNAs (paRNAs) with lengths of 200–500 nt and which do not overlap the adjacent mRNA by more than 300 nt, leaving 48 genes. Step 4: Any of the paRNAs that showed any sequence homology to known mRNAs in the National Center for Biotechnology Information (NCBI) Blast assay was excluded leaving us with 11 genes and paRNAs. (**B**) Summary of the deep sequencing data analysis results. The 11 genes are depicted after all data analysis and their matching paRNAs. The black arrow represents the paRNA while the red ones represent the corresponding mRNA. PaRNA-marked coordinates are relative to mRNAs’ TSS of the mRNA marked as +1. The blue rectangle defines genes that paRNA is overexpressed in melanoma in comparison to control, according to deep sequencing results. All of the paRNAs are sense oriented to their corresponding promoters.

Flow chart summarizing the process of the paRNAs sequences’ selection for further study is depicted in Figure 1A. Our initial screen was for ≈200–500 nt long sequences that match putative gene promoters, defined empirically as sequences that are 2000 nt upstream to known TSSs and no more than 300 nt downstream from the TSSs [20,21]. To address this, we annotated the sequences to the human normal TSS database available at DataBase of Transcriptional Start Sites (DBTSS, [22]) [23,24]. Using this sequencing annotation, we received more than 10 million reads mapped to over 360,000 sites (overall, we mapped ≈7.3 × 10^6^ reads to these sites in normal melanocytes and ≈3.1 × 10^6^ reads in melanoma). We next asked whether we could identify paRNAs whose expression differs between melanoma cells and normal melanocytes and which are mapped to promoters of genes whose expression is known to differ between normal melanocytes and melanoma. To address this question, we crossed our data with the list of genes known to be altered in melanoma relative to normal melanocytes according to [25].

Applying this additional sorting, we found ≈36,000 reads in paRNAs adjacent to 983 genes whose expression differs in melanoma compared to normal melanocytes. We then looked only at paRNAs that were differentially expressed in melanoma versus normal melanocytes and extracted from this list paRNAs that were overlapping with the adjacent gene by less than 300 bases and with a total length of no more than 500 bases. This left us with a total of 49 paRNA sequences which are expressed differentially in melanoma; 37 paRNAs’ expression was higher in normal melanocytes and 12 paRNAs’ expression was higher in melanoma (Table S1 supplementary data). In order to make sure that we identified true paRNAs and not mRNAs, each of the 48 sequences that showed any sequence homology to a known mRNA in the National Center for Biotechnology Information (NCBI) Blast assay [26] was excluded, thereby leaving us with 11 genes and paRNAs. The expression of 7 paRNAs was higher in normal melanocytes and the expression of 4 paRNAs was higher in melanoma cells (Figure 1B, marked in Table S1 supplementary data). All 11 paRNA sequences were of the sense strand and we did not detect any antisense reads.

### 3.2. PaRNA Detection and Correlation to Their Corresponding Gene Expression

**Figure 2:**
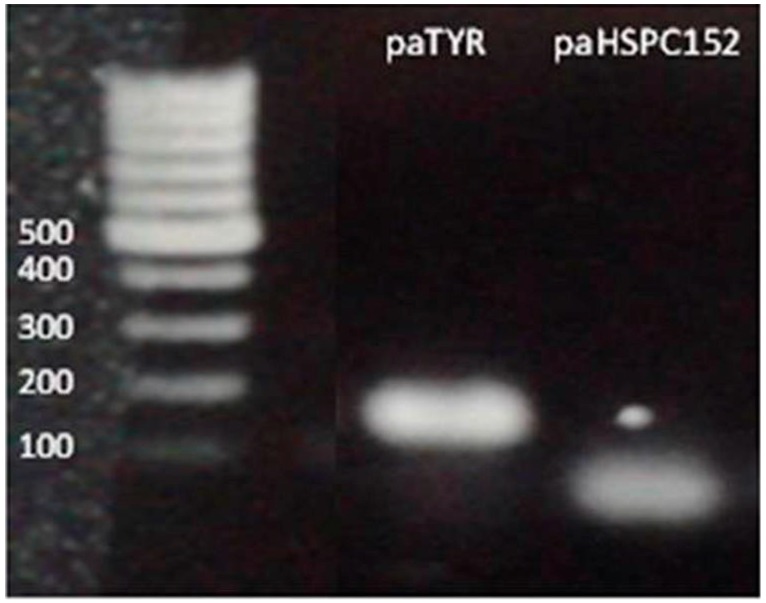
PCR products were detected under UV and were purified from the gel. Each band was sent for sequencing analysis and results were BLAST tested to verify their match to the paRNA.

Our deep sequencing data was based on comparison of one melanoma cell line to normal melanocytes. In order to better understand the role of these paRNAs in regulating the expression of their adjacent genes, we performed qRT-PCR assays of the paRNAs and their putative corresponding genes in multiple melanoma or melanocyte samples. From the deep sequencing results we could see that the expression levels of the paRNAs are low, as only a few reads were detected for each paRNA (Figure 1B). Therefore, in order to confirm that we were amplifying the paRNA, all samples were treated with DNase and the RT-PCR product was extracted from the gel and sequenced. Finally, we were successful in the reliable detection of paRNAs corresponding to HSPC152 and to the TYR both by RT-PCR of the genes (Figure 2) and by sequencing of the PCR products (data not shown).

**Figure 3:**
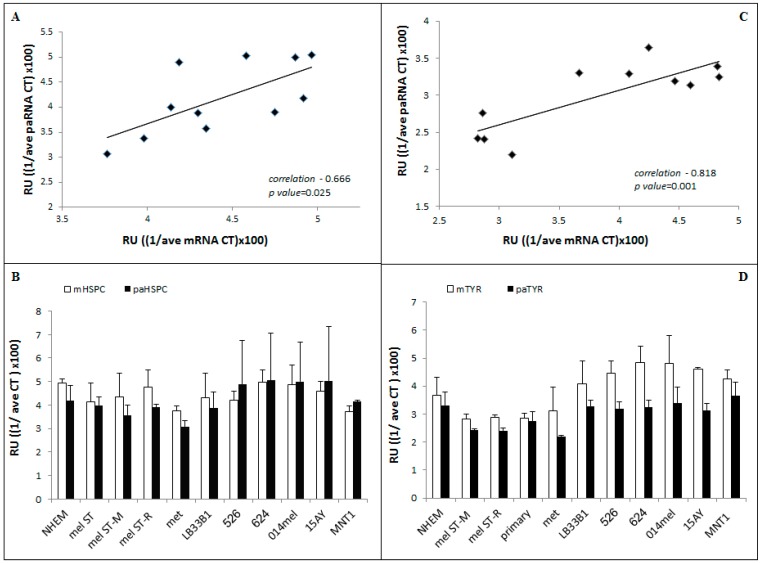
(**A**) HSPC152 mRNA (mHSPC) and paRNA (paHSPC) correlation of expression in all the tested samples and in normal melanocytes and melanoma cell lines and biopsies. (**C**) TYR mRNA (mTYR) and paRNA (paTYR) correlation (**B**–**D**). For each sample, mRNA/paRNA levels were measured using qRT-PCR (using either 1 or 2 µL of cDNA product, respectively). The graph represents the average values of ((1/ave Ct)x100) of at least three repeats per cell line or sample. All qRT-PCR results were analyzed using the same threshold (**B**) HSPC152 mRNA (mHSPC) and paRNA (paHSPC) and (**D**) TYR mRNA (mTYR) and paRNA (paTYR). Correlation calculation was done using the Pearson correlation coefficient in the statistic software IBM-SPSS (IBM, Armonk, NY, USA). The calculated correlation = 0.666, *p* value = 0.025. (Normal human epidermal melanocytes (NHEM), immortalized melanocytes (Mel-ST), transformed Mel-ST cells, Mel-STR or Mel-STM, metastatic melanoma biopsies (met, n = 5) and melanoma cell lines: melLB33B1, mel526, mel624, 014mel and 15AY).

Once the reliability of the qRT-PCR system was proven, mRNA and paRNA levels were quantified in several types of normal and transformed melanocytes, melanoma cell lines, and human melanoma biopsies. In each experiment, we correlated the expression of paRNA to the corresponding mRNA in the same RNA extract. The correlation between the paRNA expression and the expression of the adjacent gene was calculated for both HSPC152 and TYR. The expression of both paRNAs was positively correlated with their corresponding genes. The Pearson correlation coefficient between HSPC152 and paHSP152 was *r* = 0.666 and *p* = 0.025 (Figure 3A) and between TYR and paTYR it was *r* = 0.818 and *p* = 0.001 (Figure 3C).

In addition, we wanted to know whether there is a correlation between the paRNA expression level and the degree of malignant evolution of the tested melanocytes. We assayed their expression in NHEM, immortalized melanocytes (MelST cells) and in malignant-transformed melanocytes, i.e., MelST-R and MelST-M cells [17], in several melanoma cell lines and in primary and metastatic melanoma biopsies. We did not find a significant correlation between the expression level of the paRNAs and the degree of malignant evolution of melanocytes (Figure 3B,D).

### 3.3. PaRNA Effect on the Homologous Gene Expression

**Figure 4:**
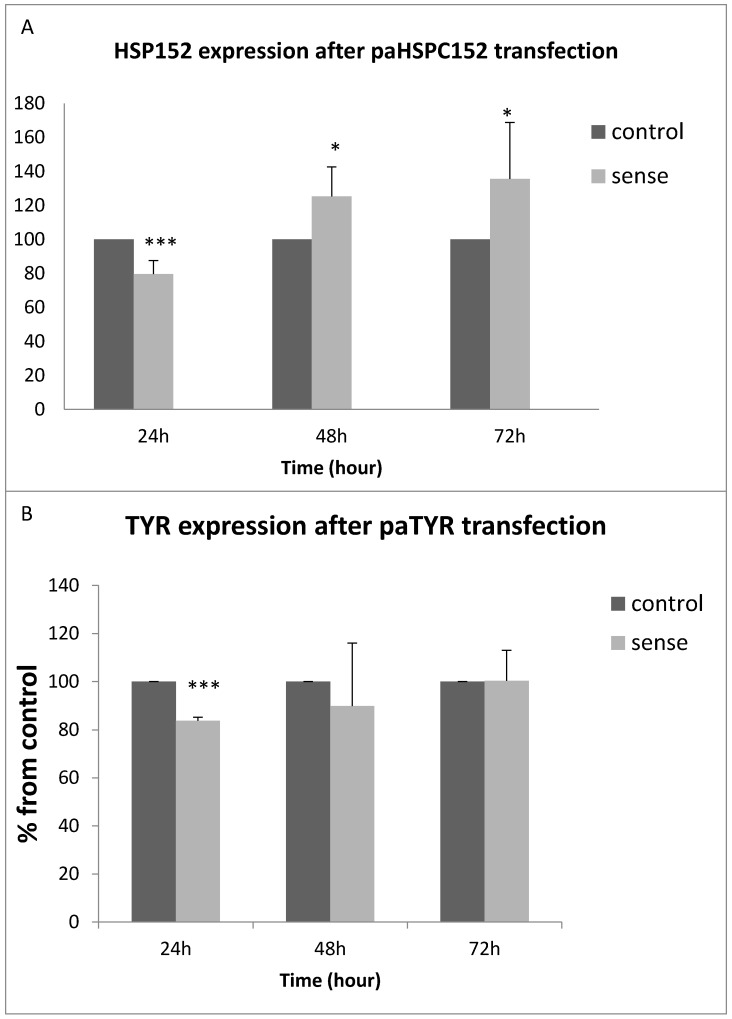
(**A**) HSPC152 gene expression 24 to 72 h after paRNA transfection in 014mel cell lines. RNA was extracted 24–72 h post transfection. Gene expression was measured using qRT-PCR. HSPC152 and TYR were measured relative to the expression of 18S RNA. The expression of HSPC152 and TYR at 24 h in untransfected cells was determined as 100%. The graph represents the mean results of at least three different experiments. * *p* value < 0.05, *** *p* value < 0.0005. (**B**) TYR gene expression 24–72 h after paTYR transfection of 014mel cells. Gene expression level was measured using qRT-PCR. The graph represents the mean results of five different experiments. *** *p* value < 0.00005.

We evaluated the effect of transient overexpression of the paRNA on the expression of its corresponding mRNA. An empty plasmid was used as a control. Following 24–72 h from transfection, RNA was extracted and gene expression was quantified using qRT-PCR and normalized relative to control non-transfected cells at 24 h. Transfection with a paHSPC152-expressing plasmid increased the level of HSPC152 at 48 and 72 h (Figure 4A). TYR expression in 014mel cells was not enhanced by its corresponding paRNA after 48 h (Figure 4B). The same results were obtained in an additional cell line, melST (data not shown).

**Figure 5:**
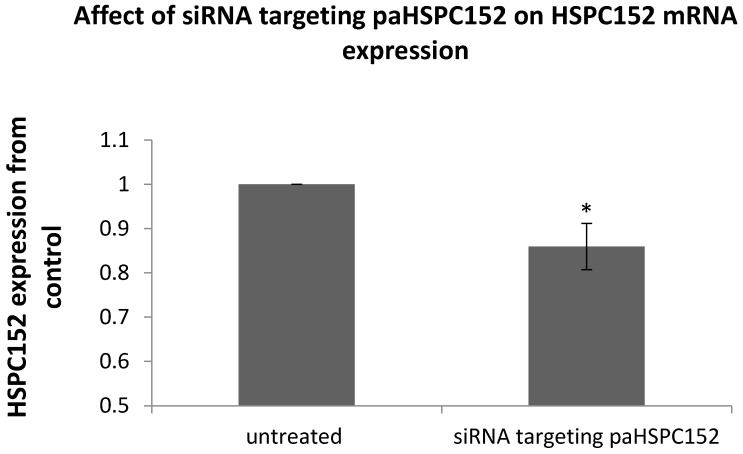
The 014mel cells were transfected with 75 nM of siRNA-targeting paHSPC152 or with 75 nM of scrambled siRNA (untreated). At 72 h post transfection, RNA was extracted and subjected to qRT-PCR with specific primers to HSPC152 mRNA. HSPC152 was normalized relative to the expression of RPLP0 RNA. The graph represents the mean results of at least three different experiments. * *p* value < 0.05.

In order to establish a causal relationship between paRNA expression and mRNA expression, we generated an siRNA targeting the sense strand of paHSPC152. This siRNA has sequence complementarity to the paRNA but not to the cognate mRNA, and can therefore interfere only with the former but not with the latter (as shown in supplementary data Figure S1). The expression of HSPC152 mRNA was decreased in cells 72 h after transfection with the siRNA (Figure 5), thereby proving that interfering with the expression of the paHSPC decreases expression from the cognate mRNA.

### 3.4. Epigenetic Modifications in Gene Promoters

It has been shown that small RNAs and lincRNAs can recruit proteins that affect the epigenetic structure of the region to which they have been recruited (review in [27]). Therefore, we asked whether overexpression of the paRNA will affect the epigenetic structure of the promoters of the studied genes.

**Figure 6:**
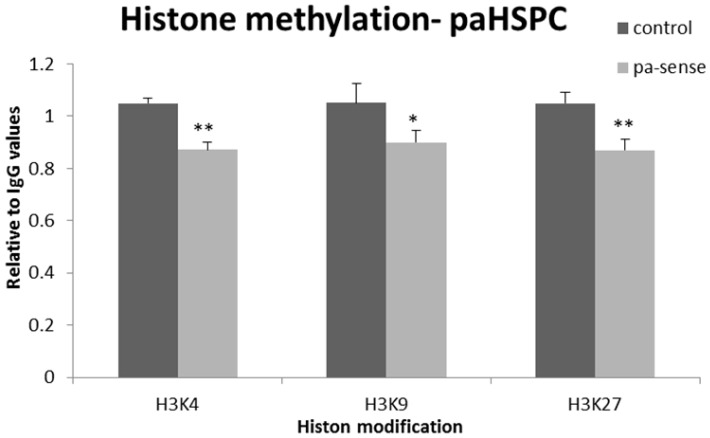
The effect of overexpression of paRNA on histone modifications of HSPC152 upstream sequences. The 014mel stably expressing paHSPC152 were subjected to ChIP assay for the three most common histone modifications on histone 3: three methylation on lysine 4, lysine 9 or lysine 27. The results were calculated as ∆Ct, and the amplification of immune-precipitated DNA with each antibody was measured relative to immune-precipitated DNA with IgG. In each, the amplification of IgG in untransfected cells was determined as 1. Graphs represent the average of three different tests. * *p* value < 0.05; ** *p*.value < 0.01.

To address this question we generated 014mel cells stably expressing sense-oriented paTYR or paHSPC152. We chose to test three different epigenetic histone modifications; H3K4me3, which correlates with active gene transcription, and H3K9me3 or H3K27me3 which are correlated with inactive chromatin (reviewed in [28,29]). The 014mel cells stably expressing an empty plasmid were used as controls. Overexpression of paHSPC slightly decreased both activating and inhibitory histone modifications of the putative HSPC152 promoter (Figure 6). Overexpression of paTYR did not affect the named histone modifications of the putative promoter region of TYR (data not shown). An additional epigenetic modification that was shown to be affected by paRNAs is DNA cytosine methylation [30]. The HSPC152 promoter region is rich with CpG islands, which might be subject to DNA methylation. Therefore, we wanted to assess whether overexpression of the paRNA will affect the DNA methylation of the named promoter. We evaluated the status of cytosine methylation of the putative promoter of HSPC152 gene by using bisulfite sequencing assay. Overexpression of paRNA did not induce cytosine methylation in 014mel cells (supplementary data Figure S2). Since the TYR promoter does not possess CpG islands, we did not test changes in DNA methylation in it.

### 3.5. The Phenotypic Effects of paHSPC152 on Melanoma Cell Lines and Immortalized Melanocytes

One of our screening criteria was for paRNAs and mRNAs whose expression differs between melanoma cells and primary melanocytes. We therefore asked whether overexpression of paRNAs of these genes will affect the cells’ malignant phenotype. We decided to focus on paHSPC152 effects, since overexpression and interference of paHSPC152 had significant effects on the HSPC152 mRNA expression. We evaluated proliferation rate, cell cycle progression and colony formation. The results of these assays in paHSPC152 transfected to 014mel cells are depicted in Figures S3–S5 in the supplement. None of these phenotypes was affected by paHSPC152 transfection. However, when the effect of paHSPC was evaluated in melST, immortalized, non-transformed melanocytes, paHSPC transfection resulted in inhibition of their proliferative activity (Figure S5) and had only a transient effect on the S phase fraction (Figure S4).

## 4. Discussion

In the present study, we searched for lincRNAs corresponding to gene promoters, referred to as paRNAs, whose expression differs between normal melanocytes and the 014mel melanoma cell line. Using deep sequencing, and after rigorous selection using several criteria for their definition (Figure 1A), we revealed the existence of 11 new paRNAs, which are sense oriented to their corresponding genes (Figure 1B) and match the previous description of paRNAs described by Preker et al. [31] and Han et al. [7].

Using qRT-PCR, we successfully verified the deep sequencing results for two paRNAs: paTYR and paHSPC152 adjacent to the TYR and HSPC152 genes and further quantified them in several melanoma cell lines and biopsies, in normal human melanocytes and in immortalized or transformed melanocytes. We found a positive correlation between the expression level of these paRNAs and the expression of their corresponding genes (Figure 3A–C), which suggests that the paRNAs are involved in the regulation of gene transcription.

We believe that both are not part of extended 5′-UTRs, even though we cannot rule out the existence of any extended 5′-UTR of those genes in the tested cell lines. However, when considering the low expression levels of the paRNAs in comparison to the mRNA expression, it is unlikely that we amplified any 5′-UTRs. 

The deep-sequencing results did not reveal any antisense RNA corresponding to the HSPC152 or to the TYR genes. Our results demonstrate that overexpression of the sense paRNA or transfection with an siRNA targeting the paRNA sense strand affected the mRNA expression in a reciprocal fashion. This suggests that the transcript we designated paHSPC is involved in the regulation of the expression of the major mRNA transcript, irrespective of whether it is part of a long 5′-UTR or an independently expressed paRNA.

The HSPC152 gene is the human homolog of the *S. cerevisiae* gene TRM112. It belongs to the transfer RNA methyltransferase gene family which catalyzes tRNA methylations [32]. It interacts with the human eukaryotic release factor-1 (heRF1) methyltransferase [33] that catalyzes the translation termination [34]. In yeast, TRMT112 homolog deletion led to a severe growth defect [35]. In Arabidopsis, inactivation of SMO2 (the TRMT112 homolog) leads to a defect in progression of cell division and organ growth [36]. Rodriguez et al. [37] suggested a genetic link between HSPC152 and breast cancer as this gene’s mRNA was amplified in 7 out of 8 cancer cell lines examined and in 26 out of 30 tumors examined.

The TYR gene encodes tyrosinase, a key enzyme in melanin synthesis. TYR has enzymatic activity that is essential to the melanogenic pathway [38]. It catalyzes conversions of tyrosine to 3,4-dihydroxyphenylalanine (DOPA) and subsequently to dopaquinone, the initial and rate-limiting step of melanin production [38]. In melanocytes, the melanogenic pathway occurs within a discrete cytoplasmic compartment, the melanosome, thus preventing the inherent cytotoxicity of the melanin intermediates DOPA, 5,6-dihydroxyindole (DHI) and 5,6-dihydroxyindole-2-carboxylic acid (DHICA), that were shown to be toxic to cells [39,40]. TYR can produce melanin precursors in the absence of TYRP1 or DCT in cells, and exogenous expression of TYR in non-melanocytic cells may cause severe cytotoxicity to cells [41]. All three melanogenic proteins, TYR, TYRP1 and DCT, affect cell growth, cell survival and cell death [42].

Overexpression of paHSPC molecules enhanced mHSPC152 expression at 48 to 72 h (Figure 4A), and siRNA targeting the paHSPC152 decreased its expression in a small yet significant manner (Figure 5). Clearly, paHSPC is not the only means by which the HSPC152 mRNA is regulated. However, our results support its function as an additional, and yet undiscovered, level of transcriptional regulation. As for the TYR gene, overexpression of the paRNA of TYR did not have any effect on TYR mRNA (Figure 4B). Those results fit our findings from the ChIP assay performed on the three most common histone modifications, where only the paHSPC152 overexpression had significant effects on the epigenetic modifications of the chromatin in 014mel sub-line (Figure 6). In addition, antisense paRNA was used to inactivate the native paRNA. The 014mel sub-line stably expressing the antisense pa-HSPC showed no effect on HSPC152 expression levels nor any changes in histone modifications in comparison to both control or sense-oriented paHSPC (data not shown).

The different effects of the two paRNAs studied here on their cognate mRNAs may imply that the mechanisms of action of paHSPC and paTYR are different. It can be hypothesized that sense paHSPC interacts with an activating polycomb protein such as the Tritorax protein complexes [43] or with the Enhancer of Zeste Homolog 2 protein that was recently shown to activate the NOTCH1 in breast cancer cells [44]. It is possible that the paHSPC attaches to the promoter area and serves as a scaffold for transcriptional activation by proteins.

Our paRNA search was based on genes whose expressions are altered in melanoma. We assumed that the alterations in the expression of the corresponding genes might affect several aspects of the malignant phenotypes—cell cycle progression and colony formation—but this was not the case: paHSPC did not affect the mentioned phenotypes in the melanoma line, while it inhibited the proliferation rate in the melST, immortalized but non-transformed melanocytes. We can conclude that although we have used a melanoma database to highlight paRNAs matching to genes with altered expression in melanoma in comparison to normal melanocytes, the HSPC152 molecule does not have a role in the malignant evolution of melanoma. This is supported by the fact that we did not find a correlation between both paRNA expression and the stage of malignant evolution of melanocytes, as shown in Figure 3, and by the lack of changes in the colony-formation assay.

Our approach to study the effect of overexpression of paRNA on its corresponding gene might be a simplified view of how these paRNAs function. Native paRNAs are transcribed upstream to their corresponding gene; hence, they operate in *cis*. Here we have shown that the overexpression of the paRNA from plasmid probably generates many paRNA molecules. In *trans*, however, paHSPC152 can still affect a gene’s expression and subsequently change a cell’s biological phenotypes. This observation is similar to findings regarding additional lincRNAs such as Evf-2 [45] and Hotair [12,46] which operate in *trans*. Further studies using our basic approach for the discovery of additional paRNAs should be applied to the study of essential regulators of gene expression in general, and in melanoma and additional cancers in particular.

## Figures and Tables

**Table 1:** Primers used in the qRT-PCR assays.

paRNA/Gene Name	Forward 5′–3′	Reverse 5′–3′
TYR	AGCACCCCACAAATCCTAACTTAC	ATGGCTGTTGTACTCCTCCAATC
paTYR	GTGGGATACGAGCCAATT	TGGCTGAGACCTATATAATACCA
HSPC152	CGTATCTGCCCTGTGGAATT	ATCAGACGCAAGTTATCGGC
paHSPC	AGCGGAGGACGACCTTTT	CATGCGAGCTCAGCAGATTG
RPLPO	CAGATCCGCATGTCCCTTCG	GCAGCAGTTTCTCCAGAGCTGG

## Supplementary Files

Supplementary File 1
