# Supplementary material for: Promoter-Associated RNAs Regulate HSPC152 Gene Expression in Malignant Melanoma
Source: Noncoding RNA. 2016 Jun 30;2(3):7. doi: 10.3390/ncrna2030007 (PMC5831909; doi:10.3390/ncrna2030007)
Supplement: Supplementary File 1 [file ncrna-02-00007-s001.docx]

**Supplementary Materials: Promoter-Associated RNAs (paRNAs) Regulate HSPC152 Gene Expression in Malignant Melanoma**

Hamutal Bonen Nitzan Kol, Noam Shomron, Raya Leibowitz-Amit, Luca Quagliata,
Thomas Lorber, Yechezkel Sidi and Dror Avni

**1S paHSPC152 sequence**

>hg18_refGene_NM_001286082 range=chr11:63841575-63842632

GGGACTGTCCCGGGAGGTGAGGCCCCCACGATTGGCGAGAAAGGTGGGCA

GCCACGAGGGCCTTCTGAAGGGATGAGCCGGGCGGGCGGAGGGGAGGGGT

CTGGCAGGCAGGAAATAAAGCGATACTCTCAGACTAGCGGGACGGGGATT

GCCATGGGGGTAGTGGGGGATGTCAGGCCCGGCCGGGCCAGCGGTCACCT

TGATTGGGGCCATGGCTGCAGCGGCTCTGCTGAAACTGCGGACCCCGCCA

GACGCCCACTCTCCTTCACTGTACCGTCTTGCTGCCGCTGCCGCAGACTG

ACCGCCGGCCCCACCGACGAGTATATAGCCCGCTGAGCGTCTCAGGGCGC

ACACGCCAGCTAGTCCCATACCCGCCCCACGGCCACTTCCACTCCGCCTC

CTGGCTGGTGCCACCGCAGCAGGCGGGGCACCGCTAGCGGCACAGCGGCG

ACACCCTGCGGAAGGCGGGCCTGGGCCGCCTCCACTGCGCAGGCGCGAGC

GGCCACCCGCAGAACAGAGCTTCCGGGACCCACGCCTCGTTTGCACTGGG

TGCTGGACAGCCGACGCAACTACAAATGGGGCGGAGCTTTCGGCACTGGA

GCAGCTAATTTGCATATAGGAATGAGGTGCGGCTCGGCTTCCATGGGCCT

AATTTACAGATAGGGCGGTATTTCTGCCCCTTAACCGAAAGTGGGATACA

GAGGACGACGGTGTTAGGCGCCTGTGTAGGAGTAAAATGTGTTTATTTTG

CATTCAACGAGAGCTCCTGCATTGCAGCTATTTTGCATATGATTTGCATC

TTACGAAGAATTTGTGGCAAAAAAAAGCTGGGCGTGCGCCGTAGGAACCT

CCTGCTGAGACGCTTCCGGTAGCGGCGCGTGACCCGACAGGTCTTTCACC

TACCTACCTCAGCTCCCACAAACACGAGAAGTTCCAGCAAGTTCGCCACT

TCCGGTTCTCCTGGCTATCCAATAGCATCGAGAGGAGCATCCCCGGAAGT

GAGGCAGCGGAGGACGACCTTTTTCCGGTTCCGGCCTGGCGAGAGTTTGT

GCGGCGAC

**Figure S1.** The promoter region of HSPC152. - The paHSPC152 sequence that does not overlap with the mRNA is marked in red. Marked in blue is the part of the paHSPC152 sequence that overlaps with the mRNA. The yellow denotes the sequence targeted by the siRNA.

**Table S1.** Identified paRNA. The positions of the transcriptional start sites (TSS) and the genes are numbered according to human Genome assembly: GRCh37. The TSS -were taken from the TSS database available at DataBase of Transcriptional Start Sites (DBTSS, [3]). The *p* value and *q* value were calculated by the CUFFDIFF differential expression method [[1](#_ENREF_1)]. The 11 genes that withstood all our criteria are marked in yellow.

| **chr** | **TSS Start** | **TSS end** | **TSS Length** | **TSS Id** | **Gene Start** | **Gene Name** | **Overlap with mRNA** | **# Reads Control** | **# Reads Melanome** | ***p* Value** | ***q*-Value** |
| --- | --- | --- | --- | --- | --- | --- | --- | --- | --- | --- | --- |
| chr10 | 99185614 | 99186112 | 498 | 127281 | 99186026 | *PGAM1* | 86 | 23 | 1 | 0 | 0 |
| chr2 | 85132480 | 85132880 | 400 | 357320 | 85132762 | *TMSB10* | 118 | 192 | 45 | 0 | 0 |
| chr5 | 122110475 | 122110877 | 402 | 51638 | 122110749 | *SNX2* | 128 | 40 | 3 | 0 | 0 |
| chr17 | 7145577 | 7146068 | 491 | 826084 | 7143737 | *GABARAP* | 176 | 60 | 6 | 0 | 0 |
| chrY | 2734781 | 2735181 | 400 | 1452833 | 2709622 | *RPS4Y1* | 216 | 27 | 0 | 0 | 0 |
| chr19 | 39466245 | 39466676 | 431 | 265536 | 39432041 | *FBXO17* | 135 | 49 | 2 | 0 | 0 |
| chr19 | 17419957 | 17420409 | 452 | 266332 | 17420336 | *DDA1* | 73 | 11 | 0 | 0.001 | 0 |
| chr11 | 88910743 | 88911143 | 400 | 613993 | 88911039 | *TYR* | 104 | 0 | 9 | 0.002 | 0 |
| chr7 | 24737713 | 24738248 | 535 | 1098749 | 24737973 | *DFNA5* | 275 | 9 | 0 | 0.003 | 0 |
| chr1 | 150131593 | 150131993 | 400 | 926386 | 150122169 | *PLEKHO1* | 232 | 8 | 0 | 0.005 | 0.001 |
| chr11 | 64084913 | 64085351 | 438 | 591544 | 64084165 | *HSPC152* | 120 | 1 | 9 | 0.005 | 0.001 |
| chr22 | 24126329 | 24126827 | 498 | 485419 | 24115035 | *MMP11* | 174 | 8 | 0 | 0.006 | 0.001 |
| chr5 | 154320510 | 154320910 | 400 | 12961 | 154320632 | *MRPL22* | 278 | 7 | 0 | 0.009 | 0.001 |
| chr1 | 24117374 | 24117811 | 437 | 228650 | 24117645 | *LYPLA2* | 166 | 6 | 0 | 0.014 | 0.003 |
| chr12 | 113344441 | 113344920 | 479 | 903811 | 113344738 | *OAS1* | 182 | 0 | 6 | 0.014 | 0.003 |
| chr11 | 67275946 | 67276366 | 420 | 1291151 | 67273967 | *CDK2AP2* | 156 | 5 | 0 | 0.025 | 0.005 |
| chr17 | 17990997 | 17991404 | 407 | 833316 | 17991282 | *DRG2* | 122 | 16 | 2 | 0.03 | 0.006 |
| chr6 | 31430662 | 31431254 | 592 | 790921 | 31430957 | *HCP5* | 297 | 4 | 0 | 0.046 | 0.008 |
| chr15 | 34633726 | 34634126 | 400 | 717480 | 34633916 | *NOP10* | 210 | 6 | 8 | 0.094 | 0.011 |
| chr11 | 64781406 | 64781806 | 400 | 583952 | 64781584 | *ARL2* | 222 | 2 | 4 | 0.132 | 0.024 |
| chr11 | 57335657 | 57336093 | 436 | 590943 | 57319127 | *UBE2L6* | 146 | 2 | 0 | 0.133 | 0.016 |
| chr9 | 97355963 | 97356466 | 503 | 665101 | 97321002 | *FBP2* | 112 | 0 | 2 | 0.157 | 0.027 |
| chr19 | 55972965 | 55973523 | 558 | 285142 | 55964345 | *ISOC2* | 84 | 1 | 0 | 0.225 | 0.026 |
| chrX | 30595733 | 30596310 | 577 | 729659 | 30576940 | *CXorf21* | 300 | 1 | 0 | 0.249 | 0.028 |
| chr17 | 8093350 | 8093750 | 400 | 1473435 | 8091650 | *C17orf59* | 214 | 1 | 0 | 0.25 | 0.028 |
| chr14 | 20937265 | 20937665 | 400 | 68394 | 20937537 | *NP* | 128 | 1 | 2 | 0.286 | 0.049 |
| chr16 | 88878217 | 88878646 | 429 | 638932 | 88875876 | *APRT* | 125 | 1 | 2 | 0.286 | 0.049 |
| chr11 | 62446333 | 62446845 | 512 | 601713 | 62443971 | *UBXN1* | 194 | 0 | 1 | 0.293 | 0.033 |
| chr12 | 48963729 | 48964129 | 400 | 866749 | 48961466 | *LALBA* | 100 | 1 | 0 | 0.317 | 0.051 |
| chr6 | 25701904 | 25702304 | 400 | 1013282 | 25652428 | *SCGN* | 104 | 1 | 0 | 0.317 | 0.051 |
| chr12 | 120765482 | 120765962 | 480 | 887799 | 120759913 | *PLA2G1B* | 110 | 1 | 0 | 0.317 | 0.051 |
| chr10 | 99446796 | 99447282 | 486 | 99759 | 99437180 | *AVPI1* | 219 | 1 | 0 | 0.317 | 0.051 |
| chr10 | 99078703 | 99079204 | 501 | 117173 | 99079021 | *FRAT1* | 183 | 0 | 1 | 0.317 | 0.051 |
| chr11 | 125773890 | 125774421 | 531 | 597581 | 125774271 | *DDX25* | 150 | 1 | 0 | 0.317 | 0.051 |
| chr19 | 49954941 | 49955388 | 447 | 263512 | 49949549 | *PIH1D1* | 150 | 19 | 12 | 0.319 | 0.051 |
| chr19 | 1095150 | 1095550 | 400 | 288852 | 1086577 | *POLR2E* | 241 | 4 | 1 | 0.593 | 0.093 |
| chr22 | 46663581 | 46664034 | 453 | 490592 | 46663860 | *TTC38* | 174 | 4 | 1 | 0.593 | 0.093 |
| chr20 | 44098264 | 44098664 | 400 | 1080343 | 44098393 | *WFDC2* | 271 | 0 | 1 | 0.317 | 0.051 |
| chr4 | 147559833 | 147560254 | 421 | 1428019 | 147560044 | *POU4F2* | 210 | 1 | 0 | 0.317 | 0.051 |
| chr20 | 3026376 | 3026806 | 430 | 823244 | 3024267 | *GNRH2* | 15 | 4 | 0 | 0.046 | 0.008 |
| chr2 | 118571967 | 118572399 | 432 | 353507 | 118572254 | *DDX18* | 145 | 87 | 31 | 0.379 | 0.06 |
| chr7 | 27196040 | 27196476 | 436 | 1164998 | 27193337 | *HOXA7* | 256 | 2 | 0 | 0.157 | 0.027 |
| chr12 | 10456736 | 10457175 | 439 | 892404 | 10457049 | *KLRD1* | 126 | 5 | 0 | 0.025 | 0.005 |
| chr1 | 22979406 | 22979853 | 447 | 257218 | 22979681 | *C1QB* | 172 | 4 | 0 | 0.046 | 0.008 |
| chr22 | 29196423 | 29196885 | 462 | 486904 | 29190547 | *XBP1* | 137 | 6 | 0 | 0.014 | 0.003 |
| chrX | 9754178 | 9754652 | 474 | 737226 | 9754495 | *SHROOM2* | 157 | 10 | 6 | 0.532 | 0.084 |
| chr5 | 174905263 | 174905696 | 433 | 26298 | 174905513 | *SFXN1* | 183 | 33 | 31 | 0 | 0 |
| chr15 | 77712737 | 77713344 | 607 | 717017 | 77713242 | *HMG20A* | 102 | 18 | 3 | 0.086 | 0.015 |

**
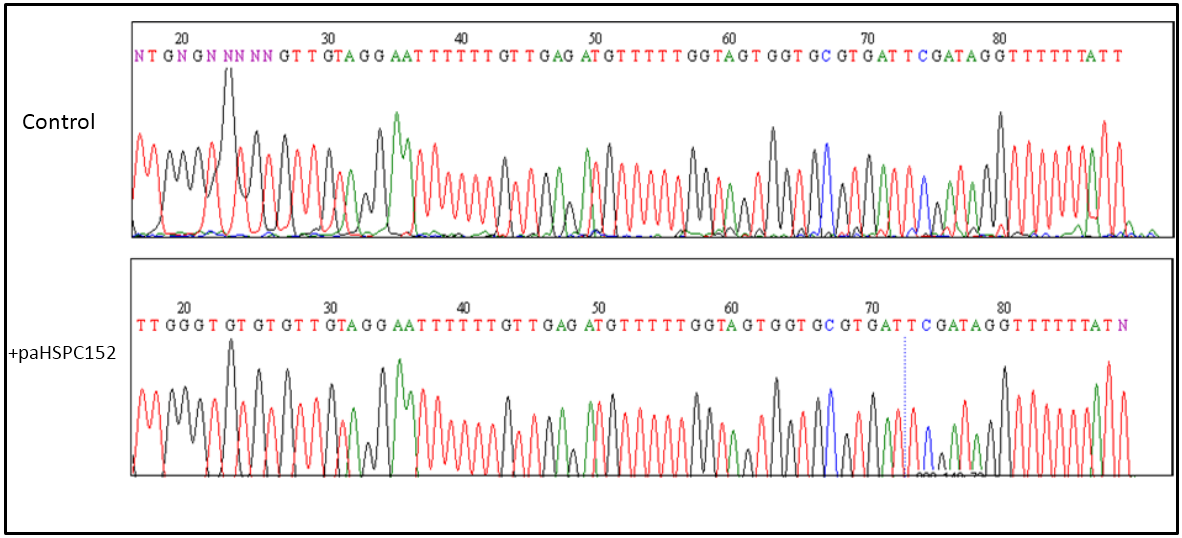
**

**Figure S2.** Effect of paHSPC152 sense or antisense overexpression on putative HSPC152 promoter methylation in 014mel cells by bisulfite sequencing in a stable line. The 014mel cells were stably transfected with a paHSPC152-expressing plasmid. Represented is a sequencing chromatogram of DNA extracted from 014mel cells. Each DNA sample was treated with bisulfite to convert unmethylated residues of cytosine into thymine, PCR amplified and then sequenced. PCR products were sequenced and analyzed by the BioEdit Sequence Alignment Editor software[[2](#_ENREF_2)].


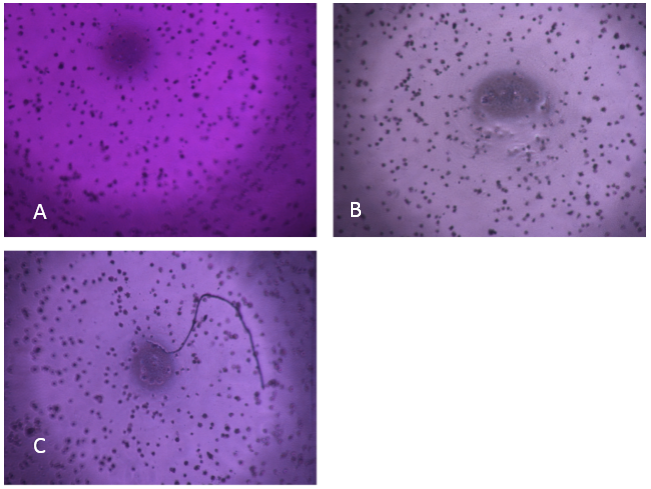


**Figure S3.** The effect of paHSPC152 overexpression on soft agar colony formation. Representative photomicrographs of colony-formation assays are shown. The assays were conducted using (**A**) 014mel; (**B**) 014mel overexpressing empty vector; (**C**) 014mel overexpressing paHSPC152. 6 × 10^3^ cells were suspended in media containing 0.6% SELECT Agar (BD Difco™ Agar, Franklin Lakes, NJ, USA) and plated on a bottom layer of media containing 1% SELECT Agar in a 96-well plate. Plates were incubated at 37 °C for 16 days before imaging. The colonies were photographed at 5× magnification.


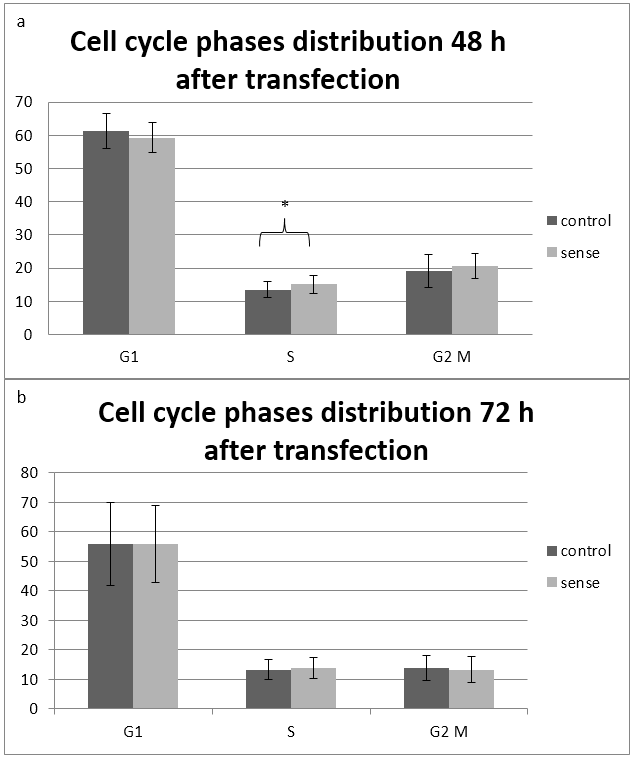


**Figure S4.** The effect of paHSPC152 overexpression on the cell cycle. MelST cell lines transfected with sense-oriented paHSPC152 were subjected to fluorescence-activated cell sorting (FACS) cell cycle analysis. paHSPC152 effect on cell cycle was tested 48 h (**a**) or 72 h (**b**) from transfection. Cells were harvested and kept in 70% ethanol in phosphate-buffered saline (PBS) solution at −20 °C until analyzed. Next, 15 min prior to FACS analysis, propidium iodide was added to the cells. The 5000 014mel or 8000 melST cells were read from each sample and analyzed using the “FlowJo” software (FlowJo, LLC, data analysis software , Ashland, OR, USA), using “24 h mock" as the baseline. G_1_—pre-synthesis phase, S—synthesis, G_2_M─ post-synthesis and mitosis phase. Graphs show the average results of at least five repeats. * = *p* value < 0.0149 as calculated by *t*-test.


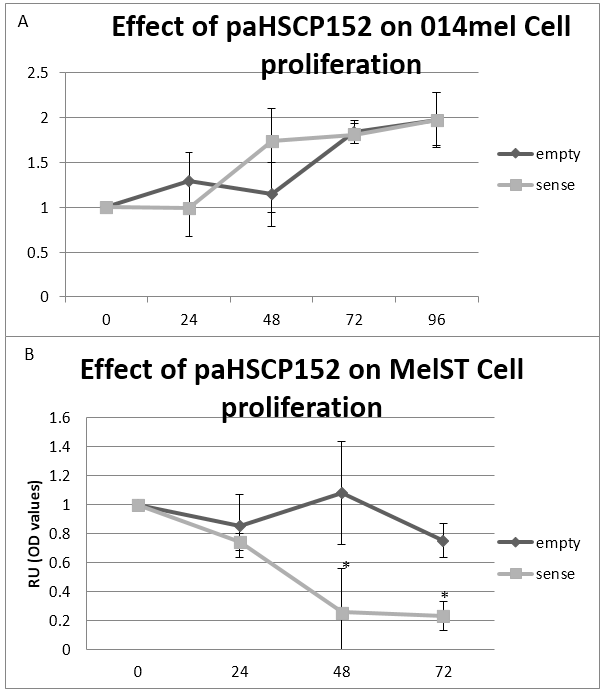


**Figure S5.** The effect of paHSCP152 on cell proliferation in (**A**) 014mel cells or (**B**) MelST cells. Cells were transfected using plasmid sense paHSPC. Proliferation was examined using the bromodeoxyuridine (BrdU) incorporation assay. ΔOD was measured for all time points, starting at time 0 (4 h from transfection) and up to 96 h. The graph represents the average of four different experiments. Graphs show the average results of at least three repeats. * = *p* value < 0.05 as calculated by *t*-test.

**References**

1. Trapnell C, Hendrickson DG, Sauvageau M, Goff L, Rinn JL, et al. (2013) Differential analysis of gene regulation at transcript resolution with RNA-seq. Nat Biotechnol 31: 46-53.

2. Hall AT (1999) BioEdit: a user-friendly biological sequence alignment editor and analysis program for Windows 95/98/NT. Nucleic Acids Symposium Series 41: 95-98.

3. http://dbtss.hgc.jp
